# Assess Quality of Life among Iranian Married Women Residing in Rural Places

**DOI:** 10.5539/gjhs.v5n4p182

**Published:** 2013-05-13

**Authors:** Seddigheh Esmaeilzadeh, Mouloud Agajani Delavar, Mohammad Hadi Aghajani Delavar

**Affiliations:** 1 Fatemezahra Infertility and Reproductive Health Research Center, Department of Obstetrics and Gynecology, Babol University of Medical Sciences, Babol, Iran; 2 Fatemezahra Infertility and Reproductive Health Research Center, Department of Midwifery, Babol University of Medical Sciences, Babol, Iran; 3 International Pardis Campus of Sari University of Agricultural Sciences and Natural Resources, Sari, Iran

**Keywords:** rural residence, Iran, women’, quality of life, WHOQOL

## Abstract

It is important how women describe their quality of life. The aim of this study was to evaluate the effects of rural residence on quality of life of the married women. The Wellness and Quality of Life Questionnaire (WHOQOL) was used to assess QOL rural residence in Iranian married women. A total of 1,140 (577 urban and 563 rural) women aged 20-45 years were selected using standard cluster sampling technique in Babol, Iran. The questionnaire with 55 items consists of five domains: physical state, mental/emotional state, stress evaluation, life enjoyment, and overall quality of life. Lower scores in three domains: physical state, mental/emotional state, and stress evaluation mean better QOL. Higher scores in life enjoyment and overall quality of life mean better QOL. Rural residences smoke more and have a lower level of education, higher level physical activity, higher level of good self reported dietary, and lower long term health problems than urban residents. After adjusting confounding variables, logistic regression indicated living in rural settings statistically decrease the probability of having worse quality of life related to physical health (OR 0.67; CI 0.50-0.91), higher life enjoyment (OR 0.44; CI 0.32-0.61), and better overall QOL (OR 0.44; CI 0.37-0.61). The results have been suggested to be useful in order to anticipate greater health care needs of the rural married women and improve their quality of life by providing more opportunities for rural women.

## 1. Introduction

The health-related quality of life (HRQL) is an important health index for different groups’ people in worldwide (Horner-Johnson, Krahn, Andresen, & Hall, 2009). The concept of the quality of life reflects individuals’ subjective and objective evaluations of both positive and negative aspects of physical health, emotional, and social functioning (Bazzichi et al., 2005; Bowling, Banister, Sutton, Evans, & Windsor, 2002; Brown, King, Butow, Dunn, & Coates, 2000; Nilsson, Parker, & Kabir, 2004). Many studies describe factors influencing HRQL among women. It has shown that more attention should be focused on improve the quality of life of women.

Since the 1980s several HRQOL instruments have been developed to measure both physical health and psychological wellbeing women (Matsubayashi, Hosaka, Izumi, Suzuki, & Makino, 2001). David Epstein produced a HRQOL questionnaire by multiple regression model with a number of variables to assess physical state, mental/emotional state, stress, life enjoyment, and overall quality (Epstein, 1996).

There are gap between the urban and rural population in Iran. In case of developed countries, rural projects are undertaken to reduce the difference between rural and urban communities.

Agricultural development and rural development are related to each other. Improving the incomes of rural families will depend crucially upon raising agricultural productivity. The relationship between urban and rural areas is changing in the world wide. Evidence from studies concluded that there are differences regarding the physical and social environment such as housing conditions, unemployment, poverty, and education level in rural and urban area. It is expected that rural women have worse health status and quality of life than urban women (Eberhardt & Pamuk, 2004; Filip & Zagorski, 2005; Probst et al., 2006; Probst, Moore, Baxley, & Lammie, 2002). However, Verma Sunil (2008) showed that total quality of life in urban area is significantly better than rural (Verma Sunil, 2008).

In the past few years, the concern for increasing the quality of life has been given to the role of place of residence in shaping individual's QOL. However, little is known about quality of life among Iranian married women or in rural women residences. The purpose of this study was to evaluate the effects of rural residence site on the perception of quality of life of the married women aged 20-45 years.

## 2. Methods and Materials

The research design of this study was a population-based cross-sectional study. The standard cluster sampling technique was used to select 1,414 married women because it allows a small number of the largest population to be studied while providing statistically valid data. Considering the geographical areas in Babol at the beginning of the study, 120 clusters (of the 100 small administrative unit urban and 300 small unit rural) were selected according to the latest census. In order to determine accurately the associated quality of life (QOL) factors with rural residence, inclusion criteria for the study were: being married for a minimum of one year, aged 20 to 45 years, being mentally sound, and having the ability to understand a questionnaire with the help of an interviewer. The sampling frame comprised by the list of census enumeration areas with population and household information from the 2009 Population Census. Each of the six districts in Babol County was subdivided into one or two cities and rural aggregations. The primary sampling unit (PSU) for this study was a ward in urban areas, or village in rural areas. Because total number of wards and villages was relatively equal, at the first stage of sampling 120 PSU (60 in urban areas and 60 in rural areas) were randomly selected. At the second stage of sampling, about 12 households per PSU on average in urban areas and about 12 households per PSU on average in rural areas were selected. Every selected the cluster was approached by the supervisors and team leaders to identify eligible women who fulfill the selection criteria after taking consent. A starting household was randomly selected in the each cluster. Each house after the first one was surveyed until the entire selected cluster had been surveyed. A total of 1,414 women seen at their home, 274 (19.4%) did not like participate in this study; thus a complete of 1,140 (577 urban and 563 rural) interviews were completed with a participation rate of 80.6 percent.

### 2.1 Data Collection

This study was approved by Babol University of Medical Sciences for ethics in medical research. Written informed consent was obtained from all participants in the study. Trained skillful personnel approached the women in the room, and carried out brief face-to-face interview to collect socio-demographic information. Following the interview, women were invited to complete QOL instruments. The following instruments were used.

A socio-demographic and clinical data form, which assesses age, age at marriage, educational level, BMI, own occupation, partner occupation, socio-economic status, own occupation, infertility.

The Wellness and Quality of Life Questionnaire (WHOQOL) instrument as a measure of the physical and psychological aspects of health-related quality of life was developed by David Epstein (Epstein, 1996). It has been translated and validated into Farsi language (Nilforooshan, Latifi, Abedi, & Ahmadi, 2006). Few recent studies have been shown that HRQOL is both valid and reliable and provides scale of QOL (Fekkes et al., 2003; Ragni et al., 2005).

The scale included the five determinants of QOL involving physical health (which includes items regarding pain, feeling of tension, energy and fatigue, sleep, incidence of colds or flu, incidence of headaches and incidence of dizziness or light-headedness), mental/emotional health (depression/anxiety and emotional control), stress (which includes items regarding general well-being, emotional well-being, significant relationships, family, health, sex life, work, and coping with daily problems), life Enjoyment (including items about overall score for the Life Enjoyment) and overall domain (which includes items regarding individual feelings relative to the quality of life) represents an assessment on QOL and health satisfaction. WHOQOL questions were scored using 5- or 7- point Likert-tpe scales.

The Physical State Scale, mental/emotional, and stress evaluation domain have a range of 10-50 and were scored using 5 point Likert-tpe scales (from 1= never/none to 5= constantly/extensive), with a lower score indicating a better QOL. The life enjoyment scale has a range of 11-55 and was scored using 5 point Likert-tpe scales (from 1= not at all to 5= extensive). The overall score of quality of life scale has 14 items scored using 5 point Likert-tpe scales from 1 (terrible) to 7 (delighted) with a range of 14-98. Higher scores in life enjoyment and overall domain of quality of life indicate a better QOL.

Rural women were referred to women living in rural areas while urban women were indicated to women living in urban area.

Fertility status and cause of infertility were assessed by self–reported questionnaire. Infertility referred to a delay in conception for least 12-months of unprotected intercourse (Schmitz, Kruse, & Kugler, 2003). The validity and reliability of questionnaire were assessed by protesting. The alpha coefficient and internal consistency of infertility was 0.80 and 0.89, respectively.

The weight of the women was recorded using digital scales to the nearest 100 grams, with the participant minimally clothed and without shoes. Height was measured with a tape measure (Craig et al., 2003). Body mass index was calculated using the formula of weight (kg)/height^2^ (m) (Higgins, 2008).

### 2.2 Statistical Analysis

All analyses were performed with SPSS (version16.0). The final multivariate model that included the lifetime infertility as dependent variables that were related to this outcome at P=0.2 in the bivariate analyses. To test the association between QOL and characteristics, stepwise multiple logistic regression was used. Odds ratio (ORs) were assessed using maximum likelihood and associated 95% CI were computed. All independent variables that met the above criteria were included in the multiple logistic regression. A P value of 0.05 or less was considered significant.

## 3. Results

Of the 1,140 women who participated, 49.4% were rural residents among study participants. The mean age of women were 33.3±7.1 years old with the slightly younger women among them living in urban settings (32.7 years old vs. 33.8 years old). Age at marriage of the women living in rural area was lower than them living in urban settings. There were more women lower education among the women living in rural area, and lower working women comparing to those in urban area. There was more self-reported good dietary, more physical activity, more smoking among women living in rural sitting. Table 1 describes the characteristics of the sample included in further logistic regression.

**Table 1:** Sample characteristics (n= 1,140)

Sample characteristics	Urban area n (%)	Rural area n (%)	P Value
Age (years)			
≤35	348(60.3)	334(59.3)	0.734
>35	229(39.7)	229(40.7)	
Age at Marriage (years)			
<19	169(29.3)	308(54.7)	0.0001
19-35	403(69.8)	251(44.6)	
>35	5(0.9)	4(0.7)	
Years of Education Completed			
<9	82(14.2)	300(53.3)	0.0001
9-12	293(50.8)	199(35.3)	
>12	202(35.0)	64(11.4)	
Own Occupation			
Housewife	435(75.4)	474(84.2)	0.0001
Worker	142(24.6)	89(15.8)	
Partner Occupation			
Manageable / Professor	214(37.1)	69(12.3)	0.0001
Intermediate	335(78.1)	398(70.7)	
Routine & Manual Occupation	28(4.9)	96(17.1)	
BMI (kg/m^2^)			
Underweight/ Normal (<25)	194(33.6)	191(33.9)	0.914
Overweight/ Obese (≥25)	383(66.4)	372(66.1)	
Level PA*			
Low	212(36.7)	81(14.4)	0.0001
Moderate	107(18.5)	81(14.4)	
High	258(44.7)	401(71.2)	
Fertility Problems			
No infertility	464(80.4)	449(79.8)	0.851
Experienced Infertility	82(14.2)	86(15.3)	
Voluntary Infertility	31(5.4)	28(5.0)	
Smoking Status			
Current or Ex-smoker	40(6.9)	85(15.1)	0.0001
Never Smoked	537(93.1)	478(84.9)	
Alcohol Use			
Yes	4(0.7)	4(0.7)	--
No	573(99.3)	559(99.3)	
Self-reported Dietary Status			
Good	542(93.9)	556(98.8)	0.0001
Bad	35(6.1)	7(1.2)	
Long Term Health Problem			
Yes	153(26.5)	112(19.9)	0.008
No	424(73.5)	451(80.1)	

* PA: physical activity

There was a statistically significant difference in the mean score in three domains (physical state, stress evaluation, and life enjoyment) between rural and urban women. We found a worse physical state, a better stress evaluation, and a better life enjoyment in women from rural area. There was no significant in the mean scores in mental/emotional state and overall quality of life between the women rural and urban area groups (Table 2).

**Table 2:** Mean wellness and quality of life scores married women accordance on residence area

	Total	Urban area (n=577)	Rural area (n=563)	P Value
Domains	Mean±SD	Mean±SD	Mean±SD	
Physical State	19.0±5.3	18.6±5.4	19.5±5.1	0.004
Mental/Emotional State	20.9±6.8	20.7±7.0	21.1±6.7	0.33
Stress Evaluation	23.5±7.8	24.0±7.9	23.0±7.7	0.030
Life Enjoyment	32.6±5.3	31.9±5.5	33.3±4.9	0.0001
Overall Quality of Life	61.5±10.1	61.1±11.5	61.9±8.4	0.142

Table 3 describes the results of logistic regression of each WHOQOL domain; five domains showed significant predictors in the model proposed (Table 3). Living in rural settings statistically decrease the probability of having worse quality of life related to physical health (OR 0.67; CI 0.50-0.91), higher life enjoyment (OR 0.44; CI 0.32-0.61), and better overall QOL (OR 0.44; CI 0.37-0.61). The women with education < 9 years had a lower probability of having worse quality of life related to physical health (OR 0.64; CI 0.43-0.93), and mental/emotional state (OR 0.64; CI 0.43-0.93), and a higher probability of better overall QOL (OR 1.82; CI 1.24-2.66). The housewife women had higher probability of having worse quality of life related to physical health (OR 1.39; CI 1.01-1.91), worse stress evaluation (OR 1.54; CI 1.12-2.12), better life enjoyment (OR 1.99; CI 1.41-2.79) and better overall QOL (OR 1.93; CI 1.83-2.52). The smoker women had lower probability to having worse stress evaluation (OR 0.62; CI 0.42-0.92).

**Table 3:** Logistic regression to determine odds ratio (OR) among women aged 20 to 45 years in each WHQOL domain (n=1,140)

Variables	Physical > 18 a OR (CI 95%)	Mental/emotional >20 a OR (CI 95%)	Stress >23 a OR (CI 95%)	Life enjoyment > 32 a OR (CI 95%)	Overall QOL > 60 a OR (CI 95%)
Residence					
Rural area	0.67(0.50-0.91)*	0.93(0.69-1.5)	0.90(0.69-1.19)	0.44(0.32-0.61)*	0.44(0.37-0.61)*
Urban area	1.00	1.00	1.00	1.00	1.00
Age at marriage (years)					
<19	0.43(0.10-1.79)	0.54(0.13-2.25)	0.46(0.11-2.01	0.24(0.04-1.27)	0.91(0.22-3.75)
19-35	0.59(0.14-2.46)	0.53(0.13-2.24)	0.60(0.14-2.61)	0.353(0.67-1.90)	1.27(0.31-5.25)
>35	1.00	1.00	1.00	1.00	1.00
Years of education completed					
<9	0.59(0.41-0.84)*	0.64(0.43-0.93)*	1.32 (0.89-1.971)*	1.18(0.79-1.77)	1.82(1.24-2.66)*
9-12	0.89(0.64-1.25)	0.78(0.55-1.09)	1.05(0.73-1.50)	1.20(0.84-1.73)	1.56(1.11-2.19)
>12	1.00	1.00	1.00	1.00	1.00
Own occupation					
Housewife	1.39(1.01-1.91)*	0.99(0.72-1.37)	1.54(1.12-2.12)*	1.99(1.41-2.79)*	1.83(1.33-2.52)*
Worker	1.00	1.00	1.00	1.00	1.00
Partner occupation					
Manageable/professor	1.22(0.77-1.94)	1.10(0.69-1.75)	1.07(0.67-1.70)	0.55(0.34-0.91)*	0.70(0.44-1.11)
Intermediate	1.05(0.70-1.57)	1.06(0.71-1.58)	0.96(0.64-1.43)	0.59(0.38-0.91)*	0.98(0.65-1.48)
Routine & manual occupation	1.00	1.00	1.00	1.00	1.00
Smoking status					
Current or ex-smoker	1.6(0.72-1.47)	0.89(0.60-1.31)	0.62(0.42-0.92)*	1.12(0.82-1.54)	1.43(0.95-2.14)
Never smoked	1.00	1.00	1.00	1.00	1.00
Alcohol use					
Yes	2.04(0.44-9.40)	0.57(0.12-2.70)	0.81(0.43-1.54)	0.65(0.12-3.51)	0.74(1.18-3.14)
No	1.00	1.00	1.00	1.00	1.00
Self-reported dietary status					
Good	1.48(0.77-2.85)	2.43(1.22-4.84)*	1.40(0.73-2.68)	0.39(0.18-0.84)*	0.46(0.22-0.93)*
Bad	1.00	1.00	1.00	1.00	1.00
Long term health problem					
Yes	0.52(0.39-0.69)*	0.50(0.38-0.67)*	0.67(0.50-0.89)*	1.62(1.19-2.21)*	1.24(0.92-1.65)
No	1.00	1.00	1.00	1.00	1.00

* P <0.05.

a Median per domain.

## 4. Discussion

Our findings showed that women living in the rural area had significantly higher level of quality of life in domain of physical, lower level of quality of life in domain life enjoyment and overall QOL than the urban women.

In the current study rural residents smoke more and have a lower level of education, higher blue-collar worker, and higher level physical activity. Several Studies support these findings of the current study (Filip & Zagorski, 2005).

The study revealed the association between quality of life with education, housewife, current or ex-smoker, and partner occupation. Conversely, Bahatia et al (Bhatia, Swami, Thakur, & Bhatia, 2007) reported a significant association between level of education and quality of life while Barau et al showed education level is not associated with QOL (Barua, Mangesh, Kumar, & Saajan, 2005). Anderson and Yoshizawa (2007) demonstrated that education physical activity, body mass index, menopause, alcohol consumption were significantly with quality of life among Australian an Japanese women (Anderson & Yoshizawa, 2007). Artazcoz et al., suggested that women workers had a better health status than housewives (Artazcoz, Borrell, Benach, Cortes, & Rohlfs, 2004).

The current study, the housewife women were associated with worse physical and stress evaluation and associated with better life enjoyment and overall domain of quality of life. Smoking status was positively associated with better stress evaluation domain of quality of life. Quality of life was not associated with age at marriage of women and alcohol use.

After adjusting some confounding variables we found that Living in rural areas was associated with better physical health-related quality of life but associated with worse life enjoyment and overall quality of life. These findings are in contrast with the results described by Zagozdzon et al. (2001) showed that living in rural areas rural residence was associated with worse physical domain of quality of life and associated with better mental health (Zagozdzon, Kolarzyk, & Marcinkowski). Whereas the other studies revealed rural woman had poor health related quality of life than the urban women (Tsai, Chi, Lee, & Chou, 2004; William, Abdel-tawab, Hassan, & Mohamed, 2004).

## 5. Conclusion

There are several limitations of the study. First, it was a cross-sectional design to assess identification factors associated with increase or decrease in QOL, which limited our ability to assess causal inference. We believe that the longitudinal studies are needed to validate these findings (Bhattacharya et al., 2009). Second, we did not compare quality of care in rural and urban area. In addition, the lack of definition the relation between health care and quality of life may have resulted in a lack of comparability of results. Third, assessing health care of rural setting and relationship of access to care with health-related care is useful for planning the total health care delivery to rural women schedule.

Despite the limitation, this study has important implication to future research and programs. Our study suggests that the quality of life of rural women was better in stress evaluation and life enjoyment domains whereas QOL in urban women was better in physical domain. This may be because of relationship some factor including education, job of women, smoking, and partner occupation with quality of life. However after adjusting this confounding variables quality of life associated with rurality. Rurality was associated with better physical, poor life enjoyment and poor overall quality of life. These results strongly suggest that Iranian health system anticipate greater health care needs of the rural married women and promote their health and welfare. The community health nurse should be able improve quality of life of rural women by providing more opportunities for rural women.

## References

[ref1] Anderson D. J., Yoshizawa T (2007). Cross-cultural comparisons of health-related quality of life in Australian and Japanese midlife women: the Australian and Japanese Midlife Women's Health Study. Menopause.

[ref2] Artazcoz L, Borrell C, Benach J, Cortes I, Rohlfs I (2004). Women, family demands and health: the importance of employment status and socio-economic position. Soc Sci Med.

[ref3] Barua A, Mangesh R, Kumar H. N., Saajan M (2005). Assessment of the domains of quality of life in the geriatric population. Indian JPsychiatry.

[ref4] Bazzichi L, Maser J, Piccinni A, Rucci P, Del Debbio A, Vivarelli L, Dell’Osso L (2005). Quality of life in rheumatoid arthritis: impact of disability and lifetime depressive spectrum symptomatology. Clin Exp Rheumatol.

[ref5] Bhatia S, Swami H, Thakur J, Bhatia V (2007). A study of health problems and loneliness among the elderly in Chandigarh. Indian J of Community Medicine.

[ref6] Bhattacharya S, Porter M, Amalraj E, Templeton A, Hamilton M, Lee A. J., Kurinczuk J. J. (2009). The epidemiology of infertility in the North East of Scotland. Hum Reprod.

[ref7] Bowling A, Banister D, Sutton S, Evans O, Windsor J (2002). A multidimensional model of the quality of life in older age. Aging Ment Health.

[ref8] Brown J. E., King M. T., Butow P. N., Dunn S. M., Coates A. S. (2000). Patterns over time in quality of life, coping and psychological adjustment in late stage melanoma patients: an application of multilevel models. Qual Life Res.

[ref9] Craig C. L., Marshall A. L., Sjostrom M, Bauman A. E., Booth M. L., Ainsworth B. E., Oja P (2003). International physical activity questionnaire: 12-country reliability and validity. Med Sci Sports Exerc.

[ref10] Eberhardt M. S., Pamuk E. R. (2004). The importance of place of residence: examining health in rural and nonrural areas. Am JPublic Health.

[ref11] Epstein D. M. (1996). Network spinal analysis: A system of health care delivery within the subluxation-based chiropractic model. JVSR.

[ref12] Fekkes M, Buitendijk S. E., Verrips G. H., Braat D. D., Brewaeys A. M., Dolfing J. G., Macklon N. S. (2003). Health-related quality of life in relation to gender and age in couples planning IVF treatment. Hum Reprod.

[ref13] Filip R. S., Zagorski J (2005). Osteoporosis risk factors in rural and urban women from the Lublin Region of Poland. Ann Agric Environ Med.

[ref14] Higgins D (2008). Patient assessment. Part 1--calculation of body mass index. Nurs Times.

[ref15] Horner-Johnson W, Krahn G, Andresen E, Hall T (2009). Developing summary scores of health-related quality of life for a population-based survey. Public Health Rep.

[ref16] Matsubayashi H, Hosaka T, Izumi S, Suzuki T, Makino T (2001). Emotional distress of infertile women in Japan. Hum Reprod.

[ref17] Nilforooshan P, Latifi Z, Abedi M. R., Ahmadi S. A. (2006). Quality of Life and its different domains in fertile and infertile women. JBS.

[ref18] Nilsson J, Parker M. G., Kabir Z. N. (2004). Assessing health-related quality of life among older people in rural Bangladesh. JTranscult Nurs.

[ref19] Probst J. C., Laditka S. B., Moore C. G., Harun N, Powell M. P., Baxley E. G. (2006). Rural-urban differences in depression prevalence: implications for family medicine. Fam Med.

[ref20] Probst J. C., Moore C. G., Baxley E. G., Lammie J. J. (2002). Rural-urban differences in visits to primary care physicians. Fam Med.

[ref21] Ragni G, Mosconi P, Baldini M. P., Somigliana E, Vegetti W, Caliari I, Nicolosi A. E. (2005). Health-related quality of life and need for IVF in 1000 Italian infertile couples. Hum Reprod.

[ref22] Schmitz N, Kruse J, Kugler J (2003). Disabilities, quality of life, and mental disorders associated with smoking and nicotine dependence. Am JPsychiatry.

[ref23] Tsai S. Y., Chi L. Y., Lee L. S., Chou P (2004). Health-related quality of life among urban, rural, and island community elderly in Taiwan. JFormos Med Assoc.

[ref24] Verma Sunil K (2008). Working and non-working rural and urban elderly: Subjective well-being and quality of life. Indian Journal of Gerontology.

[ref25] William B. M., Abdel-tawab A. M., Hassan E. A., Mohamed O. F. (2004). Acetylator phenotyping in patients with malignant lymphomas, using caffeine as the metabolic probe. Pol JPharmacol.

[ref26] Zagozdzon P, Kolarzyk E, Marcinkowski J. T. (2011). Quality of life and rural place of residence in Polish women - population based study. Ann Agric Environ Med.

